# Genome-Wide Transcriptome and Antioxidant Analyses on Gamma-Irradiated Phases of *Deinococcus radiodurans* R1

**DOI:** 10.1371/journal.pone.0085649

**Published:** 2014-01-23

**Authors:** Hemi Luan, Nan Meng, Jin Fu, Xiaomin Chen, Xun Xu, Qiang Feng, Hui Jiang, Jun Dai, Xune Yuan, Yanping Lu, Alexandra A. Roberts, Xiao Luo, Maoshan Chen, Shengtao Xu, Jun Li, Chris J. Hamilton, Chengxiang Fang, Jun Wang

**Affiliations:** 1 Department of Science and Technology, BGI-Shenzhen, Shenzhen, China; 2 College of Life Sciences, Wuhan University, Wuhan, China; 3 Key Laboratory of Fermentation Engineering, Hubei Provincial Cooperative Innovation Center of Industrial Fermentation, Hubei University of Technology, Wuhan, China; 4 School of Pharmacy, University of East Anglia, Norwich Research Park, Norwich, United Kingdom; 5 Department of Chemistry, Hong Kong Baptist University, Hong Kong, China; 6 Department of Biology, University of Copenhagen, Copenhagen, Denmark; 7 King Abdulaziz University, Jeddah, Saudi Arabia; National Taiwan University, Taiwan

## Abstract

Adaptation of *D. radiodurans* cells to extreme irradiation environments requires dynamic interactions between gene expression and metabolic regulatory networks, but studies typically address only a single layer of regulation during the recovery period after irradiation. Dynamic transcriptome analysis of *D. radiodurans* cells using strand-specific RNA sequencing (ssRNA-seq), combined with LC-MS based metabolite analysis, allowed an estimate of the immediate expression pattern of genes and antioxidants in response to irradiation. Transcriptome dynamics were examined in cells by ssRNA-seq covering its predicted genes. Of the 144 non-coding RNAs that were annotated, 49 of these were transfer RNAs and 95 were putative novel antisense RNAs. Genes differentially expressed during irradiation and recovery included those involved in DNA repair, degradation of damaged proteins and tricarboxylic acid (TCA) cycle metabolism. The knockout mutant *crtB* (phytoene synthase gene) was unable to produce carotenoids, and exhibited a decreased survival rate after irradiation, suggesting a role for these pigments in radiation resistance. Network components identified in this study, including repair and metabolic genes and antioxidants, provided new insights into the complex mechanism of radiation resistance in *D. radiodurans*.

## Introduction

*Deinococcus radiodurans* R1 is a bacterium that has the remarkable ability to tolerate high doses of radiation. It is one thousand times more resistant to ionizing radiation (IR) than humans and thirty times more resistant than *Escherichia coli* [1]. Radiation harms organisms by causing various forms of direct DNA damage including double-strand breaks (DSBs) that affect both strands of DNA and lead to the loss of genetic material [2]. The radioresistance mechanism of *D. radioduran*s does not help it avoid DNA damage, because DSBs are formed at the same rate as in *E. coli* [3]. However, *D. radiodurans* has a more efficient repair system for DNA damage, which includes double strand scission [4]. Ionizing radiation also generates reactive oxygen species (ROS), such as superoxide and hydroxyl radicals, which are chemically reactive molecules that damage cell structures [5] and also indirectly lead to DNA damage. In fact, only 20% of DNA damage is directly caused by radiation, while the remaining 80% is indirectly caused by ROS [6].

To help control ROS levels, *D. radiodurans* has a natural ROS scavenging system composed of enzymatic antioxidants, such as catalase, peroxidase, and superoxide dismutase (SOD), and non-enzymatic antioxidants, such as intracellular manganese (Mn(II)), pyrroloquinoline quinone (PQQ) and carotenoids [7]. Oxidative stress, due to an upset in the ROS balance can cause significant damage to proteins, lipids and DNA, leading to various metabolic defects, ageing, mutagenesis and even cell death [8].

The radiation resistance mechanisms of *D. radiodurans* have been categorized into three parts: 1) cellular cleansing, where oxidized nucleotides are degraded by hydrolases and damaging components are exported out of the cell; 2) antioxidant defenses, consisting of the ROS scavenging system including SOD, catalase, manganese and carotenoids; and 3) DNA repair, during which base and nucleotide excision repair and extended synthesis-dependent strand annealing and homologous recombination are active [9]–[11]. Genomics and proteomics have been used extensively to study radiation-induced molecular events. A number of bacterial genes and proteins of *D. radiodurans* have been shown to enhance the organism's ability to survive different kinds of environmental stresses [12], [13]. Previous transcriptome studies have focused on cellular recovery after exposure to irradiation, showing that induction and repression of radiation-responsive genes occurred in a time-dependent manner [14], [15]. At present, however, very little is known about the immediate transcriptome and related metabolic pathway response during γ-irradiation exposure. Here, we extend the analysis of strand-specific transcriptome profiling in the radioresistance of *D. radiodurans* using next-generation sequencing technology (ssRNA-seq). Under high dose irradiation, many *D. radiodurans* R1 RNAs were differentially regulated, including those involved in DNA repair and TCA cycle metabolism, as well as many non-coding RNAs (ncRNAs). The wild type and *crt*B knockout mutant strains were examined at various stages of γ-radiation to reveal the relationship between metabolic phenotype and irradiation resistance using a metabolomics approach.

## Materials and Methods

### Strains and Culture Conditions

All the strains were obtained from the China Center for Type Culture Collection (CCTCC). *D. radiodurans* R1 wild type and mutant strain were grown at 30°C in TGY Broth (0.5% tryptone, 0.1% glucose, 0.3% yeast extract). Cells were cultivated to late exponential stage (A_600_∼1.0) and 2.5 ml cell culture was then subjected to ^60^Co radiation with a continuous dose rate of 1000 Gray/h at room temperature. The control culture cells were not treated with radiation. The untreated control samples (DC) were taken at 0 h, and the radiated cell samples were taken at 1 h (D1) and 3 h (D3) during the radiation treatment. The recovery cell samples (DR) were incubated at room temperature and then harvested 1 h after radiation. Biological triplicates were harvested for each treatment (DC, D1, D3 and DR). These samples were immediately frozen in RNAlater (Qiagen) and stored at −80°C for RNA and metabolite analysis. The survival rates of the wild type and *crtB* mutant during ionizing radiation treatment were determined by the Plate Counting Method. The survival curve was fitted to the Linear-Quadratic Model 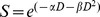 where S represented the survival rate, α and β indicated constants, and *D* was the radiation dosage) [16]. All chemicals were of reagent grade or higher, manufactured by Fisher Biosciences, except as noted.

### Construction of the *crtB* Mutant

The *crtB* mutant was constructed in *D. radiodurans* R1 by single-crossover homologous recombination as previously described by our reports [17]. Chromosomal DNA was isolated from *D. radiodurans* R1 as described by Earl *et al* [18]. PCR primers *crt*B-F (5_-TATCCATTATCGCAACTGTTTTCGC-3) and *crt*B-R (5-GTATAGTGACAGGCCGTATTCGTCG-3) were used to PCR amplify a 516 bp internal fragment of the target genes, which was thencloned into pCR-Blunt (Invitrogen, Carlsbad, CA, USA). The resulting plasmid was transformed into *D. radiodurans* R1 using a previously described method [19], and transformants were selected on TGY agar plates containing kanamycin at 25 µg/ml. Integration into the target gene in the chromosome of *D. radiodurans* R1 was confirmed by PCR and sequencing.

### RNA Isolation, Synthesis of cDNA, and Library Construction

RNA isolation, purification and cDNA synthesis were performed as follows. For all irradiated and control samples, 200 µl culture was harvested by centrifugation and washed with TE solution (pH 8.0). The pellet was resuspended in 100 µl TE solution and lysed with with lysozyme (6 mg/ml). Total RNA was then extracted using Trizol reagent (Invitrogen) and purified by chloroform and isoamyl alcohol (25∶24, v/v). After precipitation with isopropanol, total RNA was resuspended in RNase-free water, and the integrity was analyzed using Agilent Bioanalyzer 2100 (Agilent technologies). DNase I was used to digest residual DNA and the Ribo-Zero(TM) rRNA removal kit (Epicentre Biotechnologies, Madison, WI, USA) was used to deplete 16S and 23S rRNA from total RNA by oligonucleotide hybridization-mediated selective capture following the manufacturer's instructions. Fragmentation reagent (Invitrogen) was used for the fragmentation of mRNA by incubating 10 min at 90°C.

Random hexamers were used to synthesize the first strand of cDNA as follows: incubation at 25°C for 10 min, 42°C for 40 min, and 70°C for at least 15 min. The reaction was then purified with Ampure XP (Invitrogen) magnetic beads according to the instructions of the manufacturer. The second-strand cDNA was synthesized using buffer, dATPs, dGTPs, dCTPs, dUTPs and T4 DNA polymerase. After removing dNTPs, end-repair was performed at 20°C for 30 min with Klenow polymerase, T4 DNA polymerase and T4 polynucleotide kinase. A pair of Illumina PE adapters were added to cDNA templates with T4 Quick DNA ligase by incubating at 20°C for 20 min after a single 3′ adenosine was added to the cDNA using Klenow exo- and dATP. The Uracil-N-Glycosylase (UNG) enzyme was used to degrade the second-strand cDNA at 37°C for 20 min, and the reaction was purified with Ampure XP magnetic beads. Libraries were amplified by 15 cycles of PCR with Phusion polymerase, and PCR products were recovered by gel electrophoresis and purified by the MiniElute PCR Purification Kit. The length and concentration of the PCR product was checked by the Aglient Bioanalyzer 2100 and qPCR, respectively. All the libraries were sequenced using Illumina HiSeq2000. The transcriptome datasets have been deposited in the NCBI Sequence Read Archive (SRA), under the accession number SRA110026.

### Real-Time Quantitative RT-PCR Validation of ssRNA-seq Data

The PCR primers were designed for ssRNA-seq validation (Table S1 in File S1). Total RNA was extracted using Trizol reagent (Invitrogen) according to the manufacturer's instructions. After treatment with DNaseI (NEB), 5 µg of total RNA was used to synthesize the oligo (dT) primed first-strand cDNA using SuperScript™ II reverse transcriptase (Invitrogen). Real-time RT-PCR was performed on the Applied Biosystems 7500 real-time PCR System. Diluted cDNA was amplified using SYBR Premix Ex Taq™ (TaKaRa). Three technical replicates were performed for each set.

### Strand-Specific RNA–Seq Analysis

All raw reads with more than 4 N base or 45 low quality base (<Q10) were filtered. All clean reads were mapped to the genome sequence of *D. radioduran*s R1 using SOAP2 (http://soap.genomics.org.cn/soapaligner.html, version 2.21) with default settings [20]. The gene expression level is calculated using RPKM method (Reads Per Kilobase per Million mapped reads), and the formula is shown as follows:
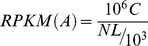



RPKM(A) is described as the expression of gene A, *C* as the number of reads that uniquely aligned to gene A, *N* as the total number of reads that uniquely aligned to all genes, and *L* as the number of bases on gene A. The RPKM method is able to eliminate the influence of different gene length and sequencing discrepancy on the calculation of gene expression [21]. Therefore, the calculated gene expression can be directly used for comparing the difference of gene expression among samples. Differential expression analysis of inter-group transcripts was performed by the DEseq package of R software. “*padj*” corresponded to p-value adjusted for multiple testing using Benjamini-Hochberg method with a significance level of <0.1 [22]. Gene reads were annotated by Gene Ontology and KEGG Pathway databases (release 59.0) using BLAST search tools (File S1) [23].

### LC-MS Based Metabolomics for Carotenoid Analysis

The carotenoids were extracted by a previously reported protocol [24]. Briefly, 2.5 ml of −48°C quenching solution (60% (v/v), methanol) was added to the collected cells. The samples were suspended and immediately centrifuged at 4000 rpm for 10 min at −20°C, and the supernatant was discarded. The cell pellets were then resuspended in 650 µl 100% methanol (−48°C), frozen in liquid nitrogen, and allowed to thaw on dry ice. The freeze-thaw cycle was performed three times in order to permeabilize the cells, resulting in the leakage of the metabolites from the cells. The cell debris was removed by centrifugation at 4000 rpm for 5 min and then 0.3 ml extraction solution (chloroform∶methanol, 2∶1, v/v) was added to the cell debris. After standing for 10 min, the mixture was centrifuged at 12000 rpm for 10 min at −4°C. The supernatant was stored at 4°C for LC–MS analysis. LC–MS data was acquired using a LTQ Orbitrap instrument (Thermo Fisher Scientific, MA, USA) set at 30000 resolution. Sample analysis was carried out under positive ion mode. The mass scanning range was 50–1500 m/z and the capillary temperature was 350°C. Nitrogen sheath gas was set at a flow rate of 30 L/min. Nitrogen auxiliary gas was set at a flow rate of 10 L/min. Spray voltage was set to 4.5 kV. The LC–MS system was run in binary gradient mode. Solvent A was 90% (v/v) methanol and Solvent B was 85∶15 (v/v) methyl tertiary butyl ether/methanol. The flow rate was 0.2 ml/min. A C-18 column (150×2.1 mm, 3.5 µm) was used for all analysis. The gradient was as follows: 85% B (0–2 min), 5% B (2–8 min), 85% B (8–12 min). Data pre-treatment was achieved using XCMS software (http://metlin.scripps.edu/download/) implemented with the freely available R statistical language (v 2.13.1). LC-MS raw data files were initially converted into netCDF format, then directly processed by the XCMS toolbox [25]. Exact molecular mass data from redundant m/z peaks was used in the online HMDB (http://www.hmdb.ca/), METLIN (http://metlin.scripps.edu/) and KEGG (www.genome.jp/kegg/) databases for metabolite searches. The metabolite name was reported when the mass difference between observed and theoretical mass was <5 ppm. The measurement of isotopic distribution was used to further validate the molecular formula of matched metabolites. The identities of the specific metabolites were confirmed by comparison of their mass spectra and chromatographic retention times with those obtained using commercially available reference standards (Figure S4 and Figure S5 in File S1) [26].

### Measurement of Thiols Using Fluorescence HPLC

Thiols were determined by labeling with monobromobimane (mBBr, Molecular Probes-Invitrogen) and analysed by high performance liquid chromatography (HPLC) with fluorescence detection as previously described [27]. Briefly, cells equivalent to 5 mg residual dry cell weight were harvested from *D. radiodurans* R1 cultures. The cell pellets were resuspended in 100 µl bimane mix (20 mM HEPES pH 8, 2 mM mBBr, 50% v/v acetonitrile) incubated for 10 min at 60°C in the dark. Reactions were stopped by the addition of 1 µl 5 M methane sulfonic acid. After centrifugation for 10 min at 12,000 g to pellet cell debris, the supernatant was pipetted out and moved to a new 1.5 ml microcentrifuge tube for HPLC analysis. The remaining cell pellets were dried overnight in an oven at 60°C.

Analytical reversed phase HPLC chromatography was performed with a HiChrom ACE-AR C18 4.6×250 mm, 5 µm, 100 Å column equilibrated at 37°C with Solvent C (0.25% v/v acetic acid and 10% methanol, adjusted to pH 4 with NaOH) as previously described [28]. Samples were eluted with a gradient of Solvent D (90% methanol) at a 1.2 ml/min flow rate as follows: 0–5 min, 0% Solvent D; 5–15 min, 0–20% Solvent D; and 15–20 min, 20–100% Solvent D, followed by re-equilibration and re-injection. Detection was carried out with a Jasco fluorescence detector with excitation at 385 nm and emission at 460 nm, and a gain of 1×. Derivatised thiols BSmB and CySmB eluted at 9.8 min and 12.3 min, respectively and were quantified by comparison with BSmB and CysmB standards of known concentration.

## Results

### Strand-Specific RNA-seq Analysis of the Gene Reads from *D. radiodurans* R1

**Figure 1:**
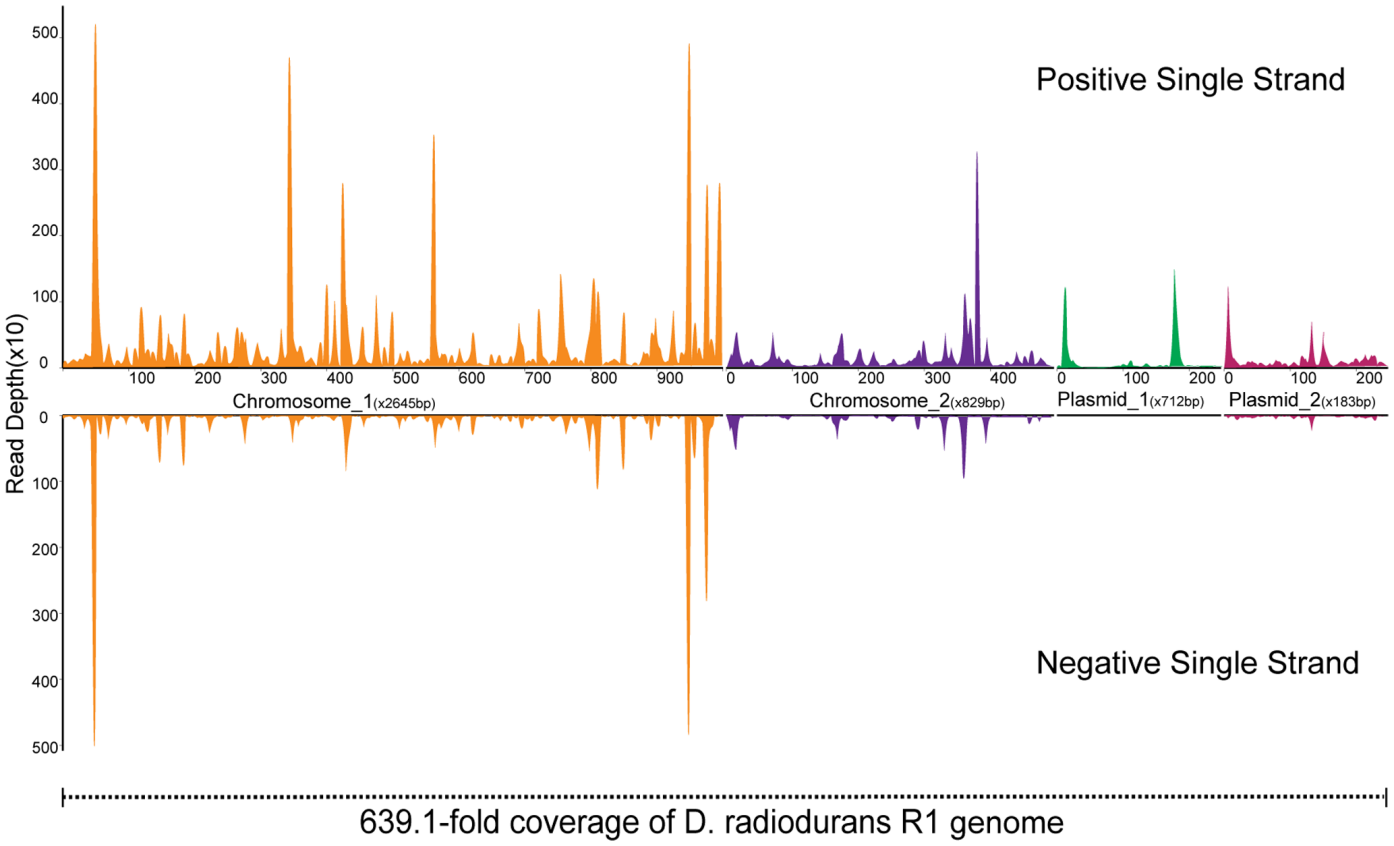
Strand-Specific RNA-seq analysis of the genes from *D. radiodurans* R1. Strand-specific coverage plot is shown. (Orange indicates the chromosome 1; violet, chromosome 2; green, plasmid 1; red, plasmid 2).

We used ssRNA-seq to characterize the genome-wide transcript abundance of *D. radiodurans* R1 before, during and after exposure to γ-rays. The accuracy of the ssRNA-seq data was verified by quantitative real-time RT-PCR analysis of 9 selected genes (Table S1 and Figure S3 in File S1). Samples were obtained at four time points to reveal the expression pattern of genes in response to irradiation. The control culture cells were not treated with radiation (DC group). The radiated cell samples were taken at 1 h and 3 h during the radiation treatment (D1 group and D3 group, respectively). The recovery cells sample were harvested 1 h after radiation (DR group). A total of ∼26 M reads were generated using Illumina sequencing technology in each sample, representing 462.7-fold coverage of the entire genome of *D. radiodurans* R1, as shown in Figure 1. The average percentage of totally and uniquely mapped genes was 87% and 81%, respectively (Table 1). Overall, 3135 of 3181 known CDSs (Protein Coding Sequence) and 50 of 68 known noncoding RNAs (ncRNAs) were detected under untreated growth conditions. These 50 ncRNAs consisted of 1 rRNA and 49 tRNAs. Ninety five *novel* putative *antisense RNAs* were further identified and annotated.

**Table 1:** Analysis of ssRNA-seq data mapped to the *D. radiodurans* genome.

	DC	D1	D3	DR
**Total Number of Reads**	26740248±944002	26396544±200481	26556410±693724	25928878±163375
**Reads Mapped**	23941320±1015264	22578095±1142993	21286440±1221411	24266685±1001548
**Percentage of Totally Mapped (%)**	89.53±1.45	85.56±5.00	80.27±6.60	93.60±4.33
**Number of Reads Mapped Uniquely**	22139498±2691744	21267205±226374	19870367±2325602	21886359±746466
**Percentage of Mapped Uniquely (%)**	82.63±7.32	80.57±0.48	75.01±10.56	84.41±2.72
**Reads Mapped to CDS**	7139367±493565	6248496±340638	4597555±454463	7005428±237476
**Reads Mapped to ncRNA**	2589875±837915	3241928±401077	4171424±555985	2772344±41207
**Reads Mapped to NC sequences**	15000131±2323609	15018709±565167	15272812±1884478	14880931±509584
**Reads Mapped to hypothetical genes**	2687128±129419	2259677±109772	1839697±191333	2808411±168006
**GC content (UNIQUE)**	0.645±0.002	0.645±0.004	0.639±0.003	0.648±0.001
**GC content (ALL)**	0.642±0.004	0.640±0.007	0.633±0.011	0.645±0.003

Means ± SD are given for each variable and each group. DC: wild type before radiation. D1: wild type with 1000 Gy radiation; D3: wild type with 3000 Gy radiation. DR: wild type 1 h after 3000 Gy radiation.

Highly expressed genes marked with high RPKM (>5000) on the positive and negative strands in the control groups were selected for analysis of the physiological functions of untreated *D. radiodurans* R1 cells. The hypothetical protein DR_0852 was the most highly expressed gene, followed by molecular chaperone *DnaK* (DR_0129) and phage shock protein (DR_1473). DR1473 (phage shock protein), a member of the signal transduction mechanism subgroup, responded strongly to irradiation as described previously [29].

### General Patterns of Expression in Response to Irradiation

**Figure 2:**
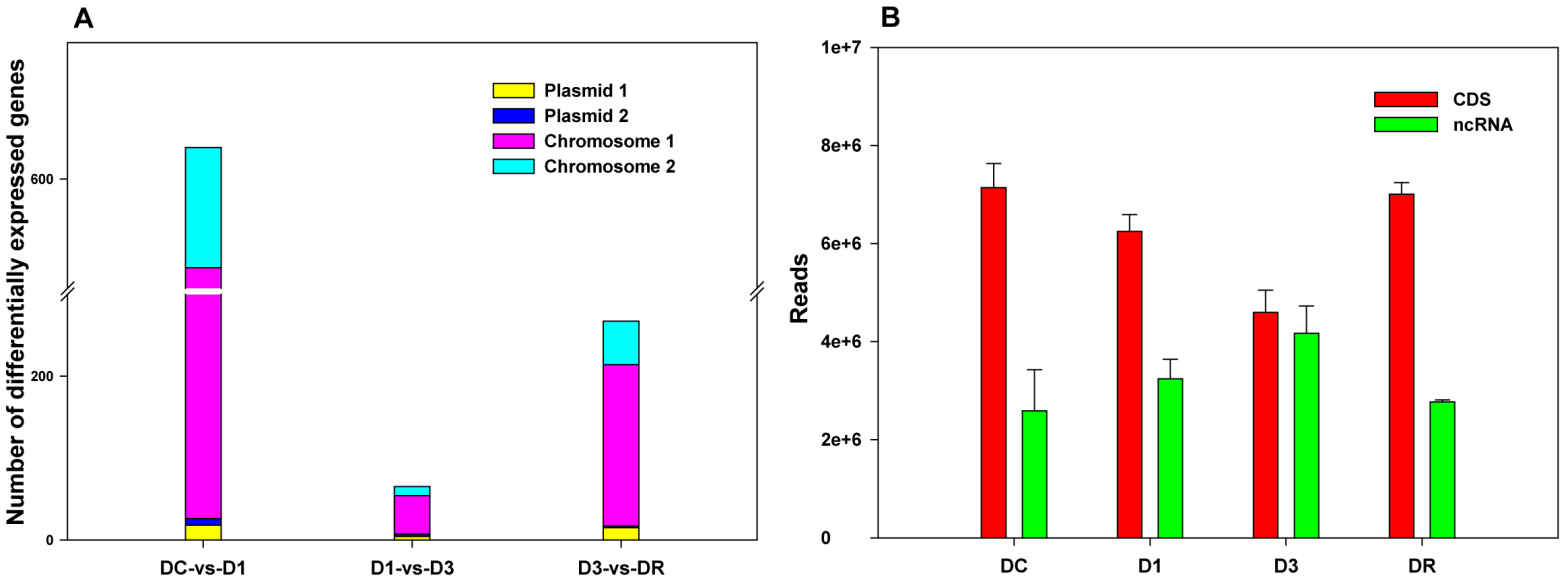
Gene expression patterns in stages of *D. radiodurans* R1 irradiation and recovery. A, The differentially expressed genes in stages of *D. radiodurans* R1 irradiation and recovery. B, The expression pattern of annotated CDS and ncRNA reads. DC: wild type before radiation. D1: wild type with 1000 Gy radiation; D3: wild type with 3000 Gy radiation. DR: wild type one hour after 3000 Gy radiation.

Interestingly, the numbers of mapped reads of CDS were lower in the irradiated (D1 and D3) group compared with the control (DC) group (Figure 2B). In contrast, the mapped reads of non-coding RNA (ncRNA) were increased in D3 group compared with DC group. This result indicated that the normal pattern of expression in *D. radiodurans* R1 was disturbed by irradiation, and returned to normal within 1 h of recovery. Using the criterion that *padj*<0.1 indicates statistically differential expression, we found there were 618 genes (19.2%) differentially expressed in the D1 group compared with DC group, and 504 of these 618 genes were located at chromosome 1. When the cells were in the late stage of irradiation treatment (D3), there were 63 genes (1.96%) differentially expressed compared to D1, which indicated that the expression pattern in response to irradiation gradually stabilized. Compared to D3, 261 genes (8.12%) showed significantly different expression levels in the recovery period. Most of the genes differentially expressed in response to irradiation and recovery were distributed at chromosome 1, followed by the chromosome 2, plasmid 1 and plasmid 2 (Figure 2A). To investigate whether genes of a particular functional group were significant contributors to radiation-induced transcription dynamics, functional categories of differentially expressed genes at each time point were generated using the functional assignments in the Gene Ontology (GO) database (Figure S1 in File S1). Almost all GO terms were intensively enriched in the stage of recovery, showing the powerful recovery capability of *D. radiodurans*. The most enriched GO term was catalytic activity, followed by binding, metabolic process and cell, showing the fundamentally physiological functions in response to irradiation. Strong variations of genes involved in cellular component and transporter activity were also observed in the early stages of irradiation and recovery, which may be important in restoring the membrane and associated ATP generation as previously reported [15]. Three genes in the GO term of antioxidant activity were significantly induced in the D1 compared with the control (DC), e.g. DR_1014 (vanadium chloroperoxidase-like protein, *padj* = 0.06), DR_A0202 (Cu/Zn family superoxide dismutase, *padj* = 0.0006), DR_A0301 (methylamine utilization protein, *padj* = 0.001). Superoxide dismutases (SOD) are enzymes that catalyze the dismutation of superoxide into oxygen and hydrogen peroxide, and thus DR_A0202 may be an important antioxidant defender in *D. radiodurans* R1 when the cells are exposed to free radicals. The sensitive response of DR_A0202 was well characterized by the dynamic transcriptome analysis, which was strongly induced in the early stages of irradiation but reduced in the later stages of irradiation and recovery.

### Dynamic RNA Profiling in Response to Irradiation using the Principal Component Analysis Model

**Figure 3:**
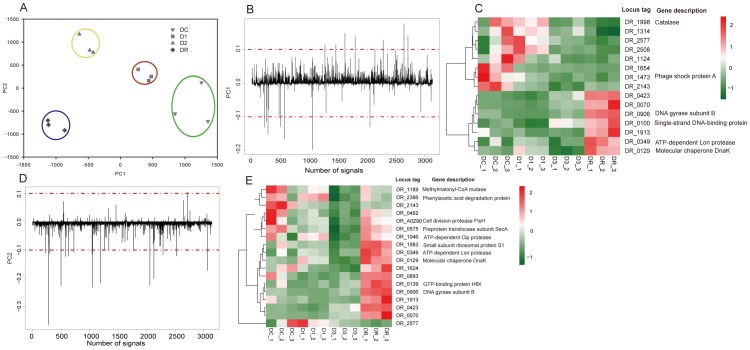
Principal component analysis of transcriptome profiling in *D. radiodurans* R1. A, the plot of the principal component 1 (PC1) versus principal component 2 (PC2) was presented. B, Loadings plot of PC1. C, Hierarchical clustering analyses of the selected genes that have a high correlation with PC1. D, Loadings plot of PC2. E, Hierarchical clustering analyses of the selected genes that have a high correlation with PC2.

To identify the main radiation-induced genes and obtain the distribution of gene expression profiling in response to irradiation, a principal component analysis (PCA) approach was applied. Briefly, Rows corresponded to the 12 samples and the columns corresponded to the RPKM of each gene. The Scores matrix provided sample scores for these expression patterns, describing the distribution of gene expression profiling between samples. Main radiation-induced genes could be identified by the Loading matrix [30]. As shown in Figure 3A, the triplicate biological samples of each group were tightly clustered. The four treatment groups were clearly separated along the direction of principal component 1 (PC1). The regular movement patterns of gene expression profiling of the four groups in response to the dose of radiation could be observed (Figure 3A). Main radiation-induced genes were selected by the loading value (>0.1 or <−0.1), and represented the high correlation coefficients between principal component 1 and the original variable (genes) (Figure 3B). Four of the 15 main radiation-induced genes (DR_0423, hypothetical protein; DR_0070, hypothetical protein; DR_0906, DNA gyrase subunit B; DR_0100, single-strand DNA-binding protein) with the same expression patterns in Figure 3C were annotated to the DNA repair system through Gene Ontology [31].

Up-regulation of these DNA repair genes appeared in the D3 and DR groups. Using the same method, 17 genes represented the high correlation between principal component 2 and the original variable (genes) (Figures 3D and 3E). The high number of up-regulated genes in the DR group demonstrated the powerful recovery capability of *D. radiodurans* R1 from γ-radiation. Fourteen of these 17 genes, including two DNA repair genes (DR_0070 and DR_0423), were strongly induced during the recovery period. DR_1046 and DR_0129 (*dnaK*) annotated as chaperone proteins could contribute to repair misfolded proteins caused by radiation [32]. DNA replication-related proteins were also induced in the recovery period, e.g. DR_0349 (ATP-dependent Lon protease), DR_1624 (RNA helicase), DR_0906 and DR_1913 (DNA gyrase subunit A). DR_0349, annotated as an ATP-dependent Lon protease, is likely to be important for cellular homeostasis by mediating the degradation of abnormal and damaged proteins, as demonstrated by the sensitivity of *E. coli lon* mutants to UV light [33]. The induced DR_0349 in the recovery stages of irradiation suggested that it may play an important role in the rapid recovery of *D. radiodurans* cells through turnover of misfolded proteins and degradation of regulatory proteins.

### DNA Repair Pathway System

**Figure 4:**
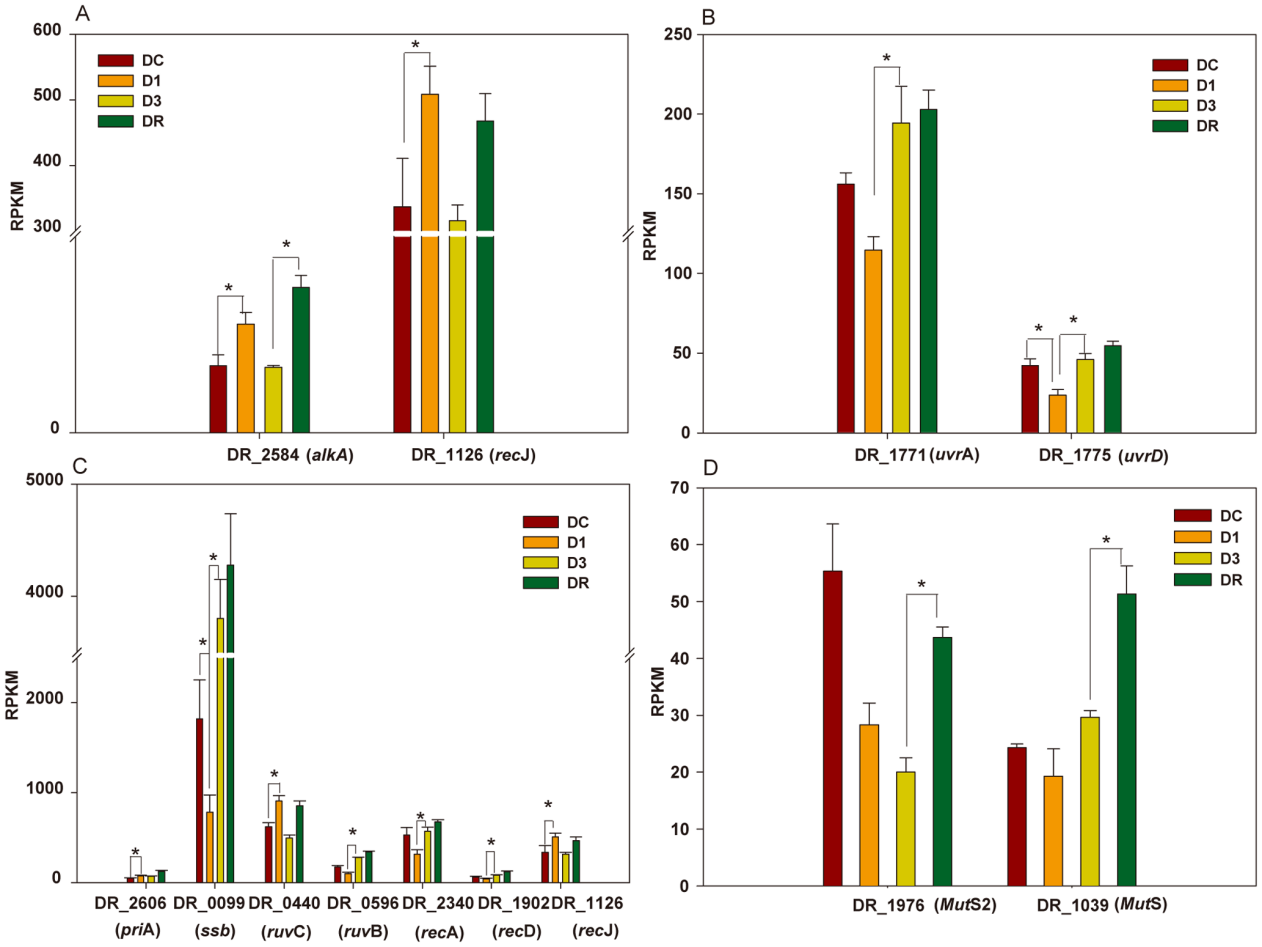
Expression patterns of selected genes for DNA repair pathway system (* *padj*<0.1). A, base excision repair. B, nucleotide excision repair. C, homologous recombination. D. mismatch repair.

The ability of *D. radiodurans* to tolerate the potentially damaging effects of ionizing irradiation can be explained by three mechanisms: prevention, tolerance and repair. A highly efficient DNA repair system including base excision repair, nucleotide excision repair, homologous recombination, and mismatch repair contributes to *D. radiodurans'* resistance to DNA damage [11]. In the present study, 2 of 11 base excision repair genes were significantly induced in D1 compared to DC, e.g. DR_2584 (*alk*A), DR_1126 (*rec*J) (Figure 4A). AlkA and RecJ may function to remove small, non-helix-distorting base lesions that are induced in the genome by radiation [34]. Although there was slightly lower expression of *alk*A and *rec*J in the later stages of irradiation (D3), it was induced again in the recovery period (DR). Nucleotide excision repair by *uvr*A and *uvr*D is a particularly important excision mechanism that can remove mutations resulting from UV-induced DNA damage. DR_1771 (*uvr*A) and DR_1775 (*uvr*D) were down-regulated in the early irradiation treatment (D1). However, at the later period of radiation and recovery, DR_1771 (*uvr*A) and DR_1775 (*uvr*D) were significantly induced (Figure 4B). Homologous recombination, encoded by *rec* pathway genes, is a type of genetic recombination in which nucleotide sequences are exchanged between two similar molecules of DNA, and is widely used to accurately repair harmful double-stranded breaks. In this study, 7 genes related to homologous recombination were significantly expressed (Figure 4C). In the late irradiation period (D3) and recovery phase, *rec*A was significantly induced. Previously, *rec*A has been detected at basal levels in untreated *D. radiodurans* cells [10], however, the elevated levels in this study suggested that extreme DNA damage and subsequent *rec*A induction was caused by the persistent irradiation. Bentchikou *et al.* reported that *rec*A activity in *D. radiodurans* is totally dependent on a functional *rec*F pathway in the extended synthesis-dependent strand annealing process (ESDSA). The rapid reconstitution of an intact genome is considered as the key ability for the extreme resistance of *D. radiodurans* [35]. The ESDSA involved in DNA double stranded break repair, followed by DNA recombination, is an important early step of genome reconstitution. The strongly induced of DR_1126 (*rec*J) at early stages of irradiation and DR_1775 (*uvr*D) at late stages of irradiation may contribute to the radio-resistance through ESDSA. However, DR1289 (*rec*Q), DR_1089 (*rec*F), DR_0819 (*rec*O), and DR0198 (*rec*R) in the *rec*F pathway were not shown to be differentially expressed under irradiation. Finally, DR_1039 (*Mut*S) and DR_1976 (*MutS*-2) involved in the methylation-dependent mismatch repair system were significantly induced in the period of recovery (DR) (Figure 4D). *Mut*S could be involved in recognizing base-base mismatches and small nucleotide insertion/deletion mispairs generated during DNA damage [36].

### Metabolic Pathway Analysis

**Figure 5:**
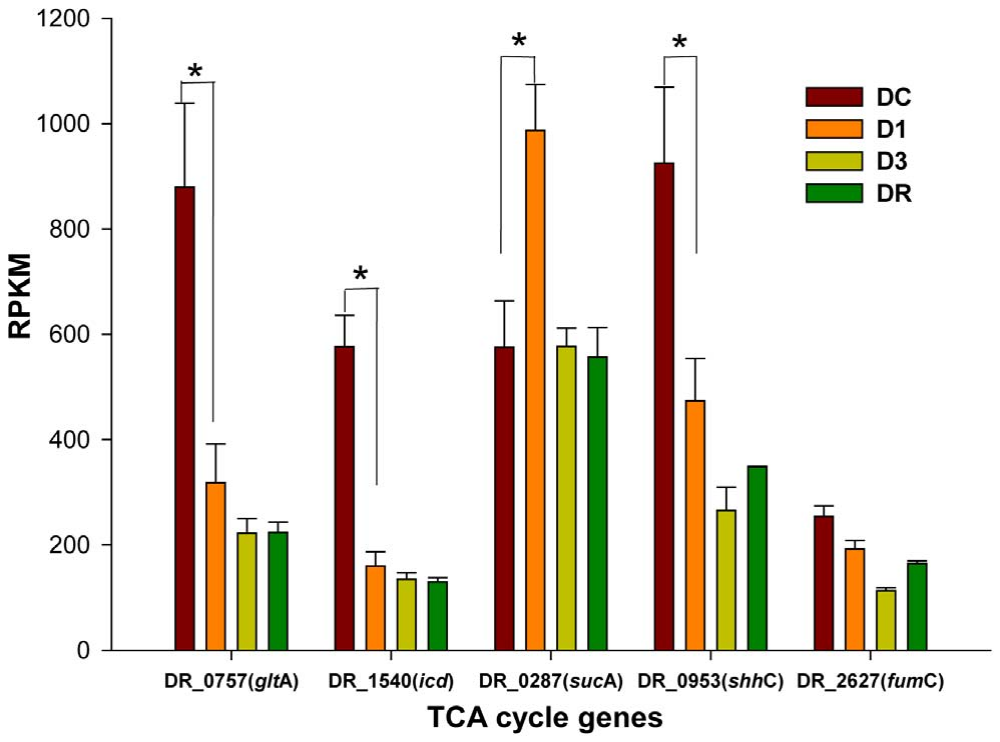
The differentially expressed TCA cycle genes during *D. radiodurans* R1 irradiation and recovery. The significant level (*padj*-value <0.1) of genes are indicated by the asterisks.

Of the 618 differentially expressed genes in the early irradiated (D1) group, 149 were mapped to the 73 metabolic pathways by iPATH2 online software (http://pathways.embl.de/iPath2.cgi#) (Figure S2 in File S1). The number of up- and down-regulated genes was 72 and 77, respectively. As shown in Figure 5, the genes DR_1540 (*icd*), DR_0757 (*gltA*), and DR_0953 (*shhC*) involved in the TCA pathway were significantly repressed during irradiation and recovery, consistent with the report of Liu, *et al* [15]. It is likely that under irradiation stress, the cell suppresses energy generation and has limited biosynthetic demands [6]. In contrast, the genes DR_0287 (*sucA*) were significantly induced at the early stage of the γ- irradiation, responding to oxidative stress caused by irradiation [37]. Those differentially expressed genes from late and recovery groups were also mapped to the 16 metabolic pathways and 72 metabolic pathways, respectively. Ten common pathways of all mapped pathways were found, e.g., sulfur relay system, sulfur metabolism, glyoxylate and dicarboxylate metabolism, carbon metabolism, biosynthesis of amino acids, cysteine and methionine metabolism, biosynthesis of secondary metabolites, microbial metabolism in diverse environments, ABC transporters and ribosome.

Bacillithiol (BSH), isolated and identified in 2009 from *Staphylococcus aureus* and *D. radiodurans*, is the α-anomeric glycoside of L-cysteinyl-D-glucosamine with L-malic acid and could function as an antioxidant [27]. BSH has been considered as a substitute for glutathione and may also be an important component responsible for the extreme ionizing-radiation resistance. A possible pathway for BSH biosynthesis in *D. radiodurans* R1 cells was predicted based on parallels to what was described in *Bacillus* species (Figure 5A) [38]. DR_1555, DR_0081 and DR_1647 were annotated as *bsh*A, *bsh*B and *bsh*C by the BLAST search tools, respectively. The expression levels of three genes were slightly reduced during irradiation (D1 and D3 groups) compared to DC, and increased again in the recovery period (DR). The concentration of BSH was determined by fluorescent HPLC, and corresponded to the gene expression data with approximately 26% lower levels of BSH at D3 (Table S2 in File S1). Interestingly, Cys levels were less affected by ionizing radiation, consistent with previous reports of the thiol levels during oxidative stress in *B. subtilis* [28]. Our data suggested that irradiation caused depletion of BSH through lower levels of biosynthesis and possibly due to oxidation of BSH due to increased cellular oxidative stressors.

*D. radiodurans* produces various carotenoids with a distinct reddish color. Carotenoids are natural pigments, generally found in plants and microorganisms. They can protect the photosynthetic apparatus from irreversible photodamage, by functioning as light-harvesters to supplement chlorophyll in scavenging free radicals. In *Deinococcus*, carotenoids have been confirmed to be a part of the ROS scavenging system [39]. We detected three important upstream carotenoid biosynthesis genes DR_0801 (*lcy*B), DR_0861 (*crt*I), DR_0862 (*crt*B), and these gene expression levels were stable under irradiation.

**Figure 6:**
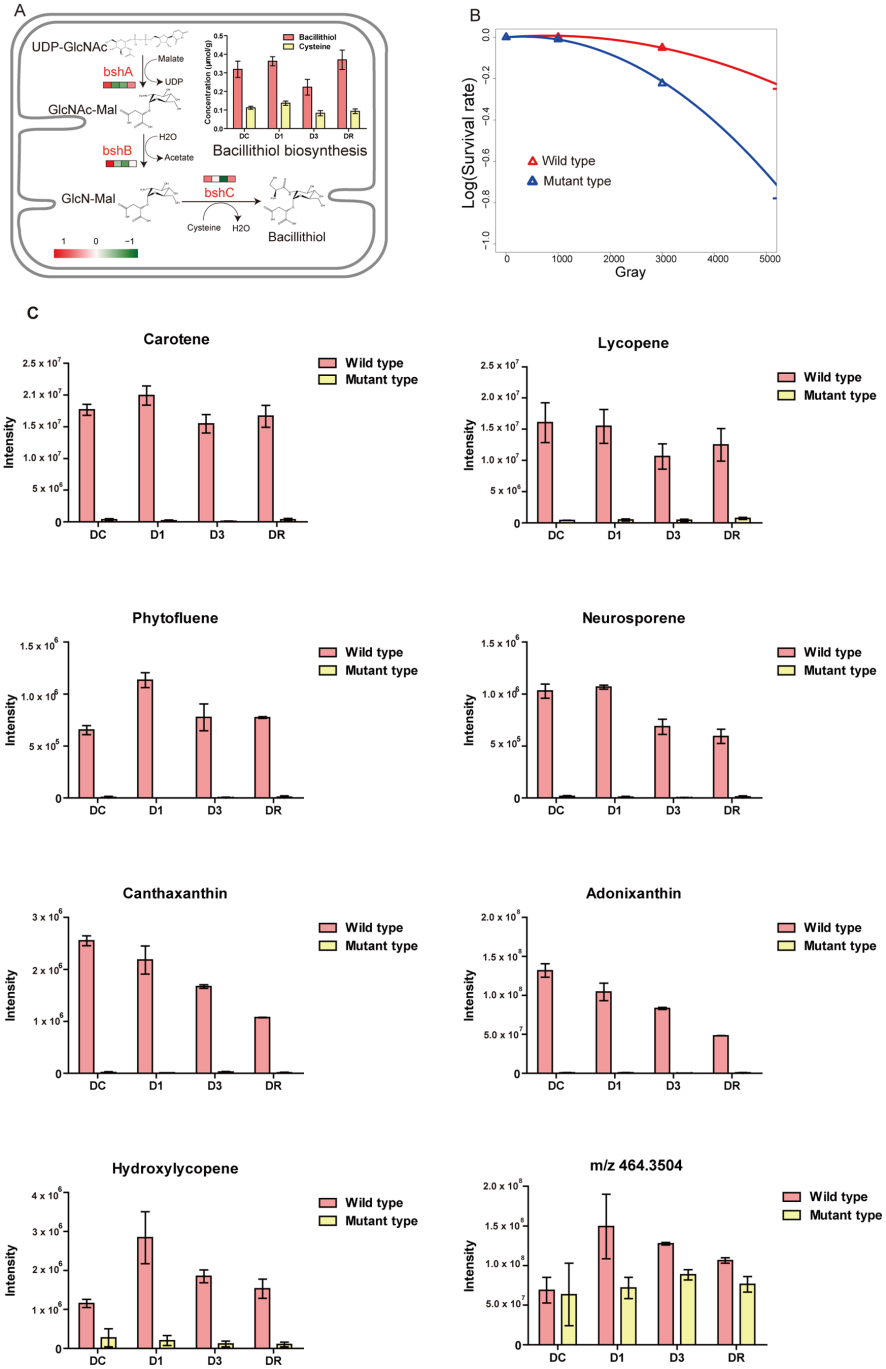
Analysis of small molecule antioxidants in *D. radiodurans* R1. A, Proposed pathway of bacillithiol biosynthesis in *D. radiodurans* R1. The enzymes involved are marked in red, and their expression level before (DC), during (D1, D3) and after (DR) irradiation are shown by the grids. The bar on the bottom left indicates the relationship between color and gene expression level. The concentration of bacillithiol and cysteine in each treatment is plotted on the top right. The error bars are standard error of the mean (SEM) values based on three biological replicates. B, the survival curves of the wild type (red) and the *crt*B deficient mutant (blue) during increasing doses of ^60^Co irradiation. An increased sensitivity to acute radiation in the *crt*B mutant is evident. C, carotenoid variation patterns in LC-MS analysis of the wild type (red) and the mutant strains (yellow).

In this study, the *crt*B (DR_0862) mutant was 30% more sensitive to acute irradiation than the wild type. *crt*B is the phytoene synthase that is involved in phytoene-derived carotenoid biosynthesis, and disruption of this gene abolishes the production of phytoene and phytoene-derived carotenoid pigments, including cryptoxanthin, carotene, neurosporene, lycopene, adonixanthin, phytofluene, hydroxylycopene (Figure 6C, Table S3 in File S1). Therefore these red-pigment carotenoids play a protective role against the lethal actions of ionizing radiation. Our data also showed that canthazanthin, adonixanthin, and lycopene had similar level at the control and early stage, and then decreased at the late and early recovery stages in the wild type strain. The accumulated gamma-irradiated dose might result in the inhibition of carotenogenesis [40].

## Discussion

This study has demonstrated that ssRNA-seq sequencing technology allows the analysis of the tanscriptome of *D. radiodurans* at the whole genome level and in a strand-specific manner. This technology provides a powerful approach to study the bacterial gene expression and mechanisms of gene regulation at the level of transcription. Transcriptome dynamics were examined in cells of two stages (immediate irradiation and recovery). The entire coverage rate of the *D. radiodurans* R1 genome was greater than 94%, which was better than previously reported [15]. The higher coverage rate facilitated the identification of genes responded to high doses of irradiation. As a functional RNA molecule that is not translated into a protein, ncRNA appears to comprise a hidden layer of internal signals that control various levels of gene expression in the metabolism of *D. radiodurans*. Our data showed the up-regulation of ncRNA expression in response to high doses of irradiation, mainly including tRNAs, which may modulate the translation of RNA to protein. In addition, many putative antisense-RNAs identified from our data may play important roles in irradiation protection in *D. radiodurans*, e.g. glycolysis pathway (DR_C0002 and DR_C0003), homologous recombination (DR_0198, *rec*R) and ABC transporter (DR_B0121, DR_B0122, DR_B0123, DR_B0124, DR_B0125, DR_2145), although the specific roles of these ncRNAs in radiation resistance need to be further validated [41]. The induced DR_0349 (ATP-dependent Lon protease) at the recovery period may contribute to the lower protein oxidation levels in *D. radiodurans*.

The highly efficient and specialized DNA repair system including excision repair, mismatch repair, ESDSA and recombination repair is considered key for the extreme radiation-resistance of *D. radiodurans*. However, the dynamic network of this system has not been deeply explored [42]. To our knowledge, there are few studies about the molecular mechanism of the synergy of the DNA repair system in *D. radiodurans*. In our study, the time series expression of genes in different DNA repair pathways showed that the response of DNA repair pathways was disparate in different stages of irradiation. Thus, these radiation-sensitive genes might be expressed at a basal level under normal growth conditions. The response of base repair was first activated in the early irradiation, while that of nucleotide excision repair was activated at late irradiation. The response of recombination repair was activated under various irradiation and recovery conditions, such as the expression levels of DR_2606 (early stage of irradiation and recovery) and the DR_2340 (*rec*A) (late stage of irradiation and recovery). DR_1172 and DR_A0065 acted as cell-growth-related genes were significantly induced in the recovery stages compared to the late stage of irradiation [15]. The believable cell-growth-related genes could not be selected from this study, because of the limited sampling time-point of recovery stage.

**Figure 7:**
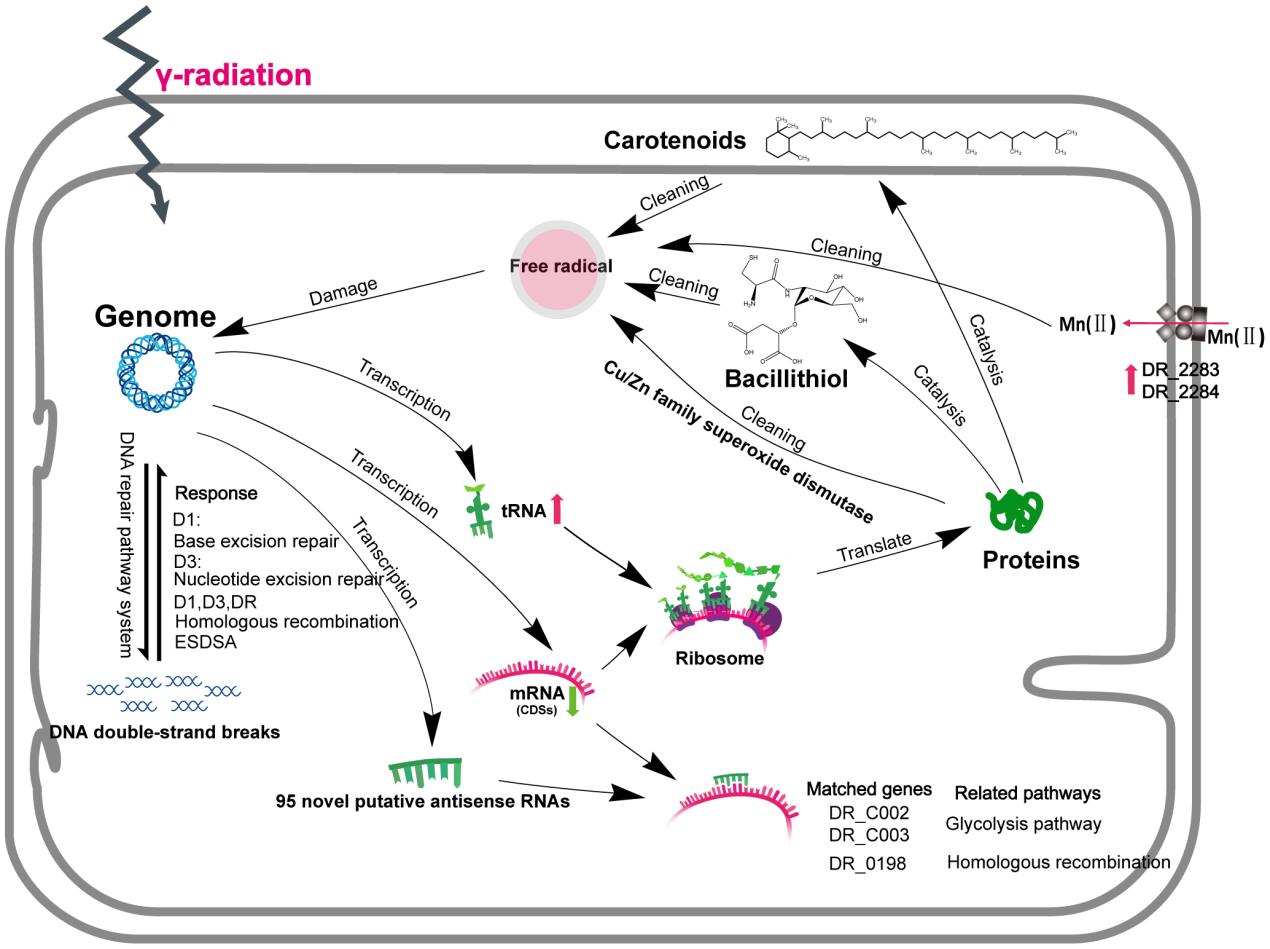
Overview of network components including genes and antioxidants in *D. radiodurans* that prevent cell damage from extreme irradiation. Red arrows and green arrows indicate the up-regulation and down-regulation, respectively. The representative *D.radiodurans* gene name is shown. The response of the DNA repair system activated in different stages of irradiation is listed. DC: wild type before radiation. D1: wild type with 1000 Gy radiation; D3: wild type with 3000 Gy radiation. DR: wild type one hour after 3000 Gy radiation.

Oxidative stress can be harmful and lead to the modification of many molecules in a cell, including DNA and proteins. To battle oxidative agents, *D. radiodurans* has developed an antioxidant system as the first line of protection. Thiol and carotenoid biosynthesis may be used to regulate the redox balance. Thiols, such as bacillithiol provide an exposed free sulfhydryl group (-SH) that is very reactive, providing abundant targets for radical attack [43]. Although our data showed that bacillithiol levels were affected by radiation, and thus may be important in recovery, confirmation of the role of BSH in radiation resistance requires the production and analysis of BSH-deficient mutants. Carotenoids protect the photosynthetic apparatus from irreversible photodamage, functioning as light-harvesters to supplement chlorophyll to scavenge free radicals. Furthermore, carotenoids have been considered as a part of the ROS scavenging system in this *Deinococcus* strain [39]. The *crt*B (DR_0862) mutant *D. radiodurans* without protection of carotenoids has shown the radiation sensitivity. Interestingly, a compound (C_28_H_48_O_5_) with a mass/charge ratio of 464.3504 and a retention time of 8.7 min was present in D. radiodurans. And the study revealed its clear correlation to radiation stress. Additionally, the knockout of *crt*B appeared to have a noticeable drop of the concentration of this compound and nearly blocked its increase during radiation (Figure 6C). The information in this study provided a clue of the biological roles of this compound in radiation resistance, as an alternative to carotenoids-based resistance. In addition to these antioxidant systems, Daly proposed that high levels of manganese could also protect proteins from the ROS produced during irradiation [9]. Our data showed that DR_2283 and DR_2284 (manganese ABC transporter permease) functioning as Mn^2+^ transporters were induced in the early stages of irradiation and recovery. An overview of components of the irradiation protection network in *D. radiodurans*, including genes and antioxidants that prevent cell damage, was summarized in Figure 7.

In conclusion, dynamic transcriptome analysis of *D. radiodurans* cells, integrated with ssRNA–seq and LC-MS based metabolomics have allowed an estimate of the immediate expression pattern of genes and metabolites in response to the irradiation. Components of the irradiation resistance network, including genes and antioxidants, provided insight into the complex and multi-layered molecular regulation mechanism. Our data suggested that the response of radiation–sensitive genes in the *D. radiodurans* could be readily triggered by an extreme irradiation environment. Functional categorization of the differentially expressed genes showed that DNA repair systems and antioxidant systems would be activated in the *D. radiodurans* cell, when the cells were treated with ^60^Co irradiation.

Furthermore, our data provided the novel view that the DNA repair system response could be activated in different stages of irradiation, not only the recovery period. The response of the radiation–sensitive gene that encodes superoxide dismutase was well characterized by our ssRNA-seq technology. This gene could be strongly induced in the early stages of irradiation, considered as an early warning signal for oxidative stress caused by ROS. Many annotated ncRNAs functioning as regulators may participate in the regulatory network in response to irradiation. LC-MS based metabolomics, a method for qualitation and quantitation of all small molecule metabolites in biological matrices, has become an important tool in the study of systems biology. Briefly, this study represented new insights in understanding the mechanism of radiation resistance in *D. radiodurans* R1, providing significant implication for radiation waste management and drug development [44].

## Supporting Information

File S1 **Table S1**, List of primers used for real-time RT-PCR validation of RNA-seq based data. **Table S2**, The concentration of bacillithiol and cysteine. **Table S3**, The annotated carotenoids compounds list. **Figure S1**, Functional categories of significantly expressed genes at each time point. **Figure S2**, The significantly expressed genes mapped metabolic pathways in the D1 compared to DC group using iPATH2 online software. **Figure S3**, Representative inter-group correlation coefficients were calculated by the Spearman Rank-Order Correlation. **Figure S4**, The representative total ion chromatogram of LC-MS based metabolomics for *D. radiodurans* R1 cell. **Figure S5**, The positive ion MS/MS fragmentation of the lycopene from wild strains extracts (top panel) and the positive ion mode fragmentation spectrum for synthetic lycopene (bottom panel).
(DOC)
