# Supplementary material for: Genome-Wide Transcriptome and Antioxidant Analyses on Gamma-Irradiated Phases of Deinococcus radiodurans R1
Source: PLoS One. 2014 Jan 23;9(1):e85649. doi: 10.1371/journal.pone.0085649 (PMC3900439; doi:10.1371/journal.pone.0085649)
Supplement: File S1 — Table S1, List of primers used for real-time RT-PCR validation of RNA-seq based data. Table S2, The concentration of bacillithiol and cysteine. Table S3, The annotated carotenoids compounds list. Figure S1, Functional categories of significantly expressed genes at each time point. Figure S2, The significantly expressed genes mapped metabolic pathways in the D1 compared to DC group using iPATH2 online software. Figure S3, Representative inter-group correlation coefficients were calculated by the Spearman Rank-Order Correlation. Figure S4, The representative total ion chromatogram of LC-MS based metabolomics for D. radiodurans R1 cell. Figure S5, The positive ion MS/MS fragmentation of the lycopene from wild strains extracts (top panel) and the positive ion mode fragmentation spectrum for synthetic lycopene (bottom panel). (DOC) [file pone.0085649.s001.doc]

# Genome-Wide Transcriptome and Antioxidant Analyses on Gamma-Irradiated Phases of *Deinococcus radiodurans* R1

Hemi Luan^1,5#^; Nan Meng^1#^; Jin Fu^1#^; Xiaomin Chen^1#^; Xun Xu^1^; Qiang Feng^1^; Hui Jiang^1^; Jun Dai^2,3^; Xune Yuan^1^; Yanping Lu^1^; Alexandra A. Roberts^4^; Xiao Luo^1^; Maoshan Chen^1^; Shengtao Xu^1^, Jun Li^1^; Chris J. Hamilton^4^; Chengxiang Fang^2^*; Jun Wang^1,6,7^*

1 Department of science and technology, BGI-Shenzhen, Shenzhen, China;

2 College of Life Sciences, Wuhan University, Wuhan, China;

3 Key Laboratory of Fermentation Engineering, Hubei Provincial Cooperative Innovation Center of Industrial Fermentation, Hubei University of Technology, Wuhan, China

4 School of Pharmacy, University of East Anglia, Norwich Research Park, Norwich, UK;

5 Department of Chemistry, Hong Kong Baptist University, Hong Kong, China

6 Department of Biology, University of Copenhagen, Copenhagen, Denmark;

7 King Abdulaziz University, Jeddah, Saudi Arabia;

* To whom correspondence may be addressed

# These authors contributed equally to this work.

Name and complete address for correspondence:

Professor Jun Wang,

Fax number: +86 755 2235 4236 (Office); Tel: +86 755 2232 5193(Office);

E-mail: wangj@genomics.cn; Address: Building No.11, Beishan Industrial Zone, Yantian District, Shenzhen 518083, P.R. China

Professor Chengxiang Fang

E-mail: cxfang@whu.edu.cn; Address: LuoJiaShan, Wuchang District, Wuhan 430000, P.R. China

**Materials and Methods**

**Quality Control and Read Mapping**

The raw sequence data were filtered with the quality control standard (N rate≥0.05 and the rate of bases (quality≤10) in a read ≥ 0.5). Furthermore, adapter or ploy-A tail sequences within raw reads was masked before mapping. SOAPaligner version 2.21v was used to align the clean reads to the NCBI *D. radiodurans* R1 genome reference for assembly, with a maximum of five mismatches and default parameters. Reads that aligned uniquely were collected for read mapping statistics and subsequent analyses. All data were calculated as Means ± SD.

**Differential Gene Expression and Enrichment Analyses**

To estimate the overall gene expression, the reads per kb per million mapped reads (RPKM) value were calculated. The correlation tests of the RPKM value among triplicate strains (DC1, DC2 and DC3) were employed to verify the experiment’s reliability and reproducibility. The differentially expressed genes between two groups in different stages were selected using an R package named DESeq, according to the default parameters that the significantly differential expression at false discovery rate (FDR) of 10% (*padj* < 0.1) adjusted by Benjamini-Hochberg multiple testing. Up- and down-regulated expressions were represented by the log_2_ (fold change) > 0 and < 0, respectively. Identification of differentially expressed genes exhibiting similar expression patterns was performed using hierarchy clustering algorithm provided by “pheatmap” package of R software. Also enrichment analyses using hypergeometric test were carried out to find out significantly enriched GO terms (*p* ≤ 0.05, with Bonferroni correction) and pathways (FDR ≤ 0.05) within the differentially expressed genes.

**Gene Annotation**

The differentially expressed genes were annotated with Gene Ontology (http://www.geneontology.org/) and KEGG (http://www.genome.jp/kegg/) databases based on the blastx alignment program (E-value ≤ 10^-5^, valid hits ≤ 5) and nr database.The highly expressed genes (RPKM value ≤ 5.0×10^3^) on both positive and negative strands and the genes generated from PCA loading results were annotated with Gene Ontology (http://www.geneontology.org/) and KEGG (http://www.genome.jp/kegg/) databases based on the blastx alignment program (E-value ≤ 10^-3^, valid hits ≤ 20) and nr database.

**Novel Transcript Prediction**

To discover new transcribed regions respectively in four groups after irradiation for different hours, we compared our assembled transcripts predicted by Cufflinks (http://cufflinks.cbcb.umd.edu/) software and annotated genomic transcripts from reference sequences. To be reported as a novel transcript, an assembled transcript must meet two requirements including the transcript must be at least 100bp away from annotated gene, and the sequencing depth is no less than 2.

**Results**

**Reliability and reproducibility of ssRNA-seq**

The Spearman correlation test was used for assessment of the correlation of biological replicates in each group. A significant positive correlation was observed that all the coefficient correlations were higher than 0.9. This result showed the good reliability and reproducibility of ssRNA-seq (Figure S3). Selected genes were validated by RT-PCR and qPCR methods. The primers used in these analyses are shown Table S1. All results as described above confirm that RNA-Seq profiling was suitable for the quantitative assessment of expressed genes.

**LC-MS based metabolomics for carotenoids analysis**

Using the optimized HPLC-MS analysis protocol and subsequent processes, such as baseline correction, peak deconvolution, alignment and normalization, a three-dimensional matrix was formed, including data filename, retention-time exact mass pair and normalized peak areas. There were 4128 retention-time exact mass pairs determined in each sample profile. The total ion chromatogram was shown in the Figure S4. As described, 7 identified carotenoids compounds were presented (Table S3). The carotenoids metabolites were identified by online databases (i.e. HMDB, METLIN) using accurate mass and isotopic distribution patterns to match the metabolites. The high-resolution ESI-LTQ-ORBITRAP data was important to note that the m/z values of metabolites were measured within a mass error of 5 ppm, allowing elemental formula confirmation. These carotenoids were detected as radical molecular ions (M^•+^), as previously described for the electrospray behavior of carotenoid species. We also verified some of the carotenoids using MS/MS analysis. For example, we verified the identity of lycopene by MS/MS analysis, which was down-regulated in *crtB* knockout mutant strains. The figure depicts the positive ion MS/MS fragmentation of the lycopene from wild strains extracts (top panel) and the positive ion mode fragmentation spectrum for synthetic lycopene (bottom panel) (Figure S5).

Table S1 List of primers used for real-time RT-PCR validation of RNA-seq based data.

| Genes | Primers (5'--3') | |
| --- | --- | --- |
|  | Forward | Reverse |
| DR_0081( *bsh*B) | CTCGTTCTCAGGTGGCGTCT | CACCAGTTTCCCGACAAATACC |
| DR_0287( *suc*A) | GCGGGTCGGAGATGGTAAA | AGGGCGTGGTCATGGAGAC |
| DR_0349 | TGATGCTCGTTTCCACAA | CCTCAAGCCCAACCAGAT |
| DR_0861 (*crt*I) | AGCAGCGTCTCGAAGGAA | GAACTCGGCTACACCCAT |
| DR_0953 (*shh*C) | GACCCACTGTGCCGAAAG | CGAGCTGAACTGGTGGATTT |
| DR_1126 (*rec*J) | GTATTTCATGCCTTTGACG | GAGCATTCTGGAACCCTT |
| DR_1771 ( *uvr*A) | TGTGGCTGGTCGTCTTCT | ACCGCTTCGTGGTGATTA |
| DR_1775 (*uvr*D) | TACACTTTGGCGTCGGGATA | GCGACCTGATTACCGAAACG |
| DR_2340 (*rec*A) | TCGCCGTAGGAGTAGAAGC | CGCCAACACCGTCAAGAT |
| 16S | TCGTGTCGTGAGATGTTGGGTT | AGACGCCTGCCGTTAAGCT |

Table S2 The concentration of bacillithiol and cysteine

| **Groups** | **Bacillithiol (μmol/g, mean±SE)** | **Cysteine (μmol/g, mean±SE)** |
| --- | --- | --- |
| DC | 0.319±0.044 | 0.112±0.008 |
| D1 | 0.362±0.024 | 0.083±0.011 |
| D3 | 0.222±0.042 | 0.083±0.014 |
| DR | 0.370±0.052 | 0.093±0.012 |

Table S3 The annotated carotenoids compounds list

| **M/Z** | **Retention time (min)** | **Adduct ion** | **ppm** | **Metabolites** |
| --- | --- | --- | --- | --- |
| 536.4365 | 9.4 | [M]^+^ | 3.10 | Lycopene |
| 538.4525 | 9.6 | [M]^+^ | 2.40 | Neurosporene |
| 540.4677 | 9.7 | [M]^+^ | 3.40 | carotene |
| 564.3951 | 7.0 | [M]^+^ | 2.84 | Canthaxanthin |
| 582.4054 | 7.0 | [M]^+^ | 3.17 | Adonixanthin |
| 542.4852 | 9.8 | [M]^+^ | 0.11 | Phytofluene |
| 554.4461 | 8.7 | [M]^+^ | 4.83 | Hydroxylycopene |
| 464.3504 | 8.7 | [M]^+^ | 0.47 | C_28_H_48_O_5_ |


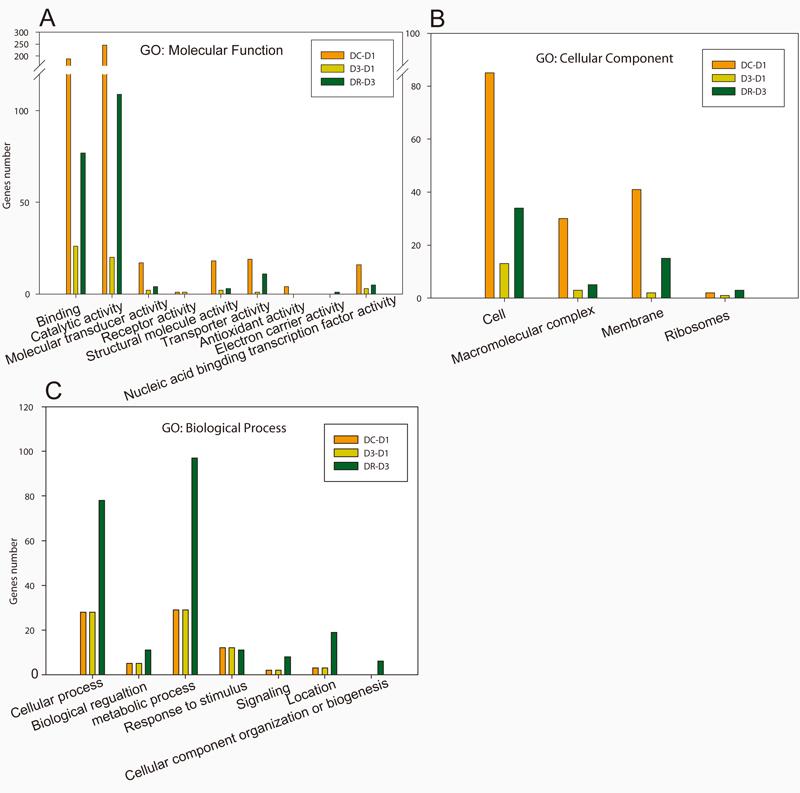


Figure S1. Functional categories of significantly expressed genes at each time point. A, Levels of molecular function. B, Levels of cellular component. C, Levels of biological process.


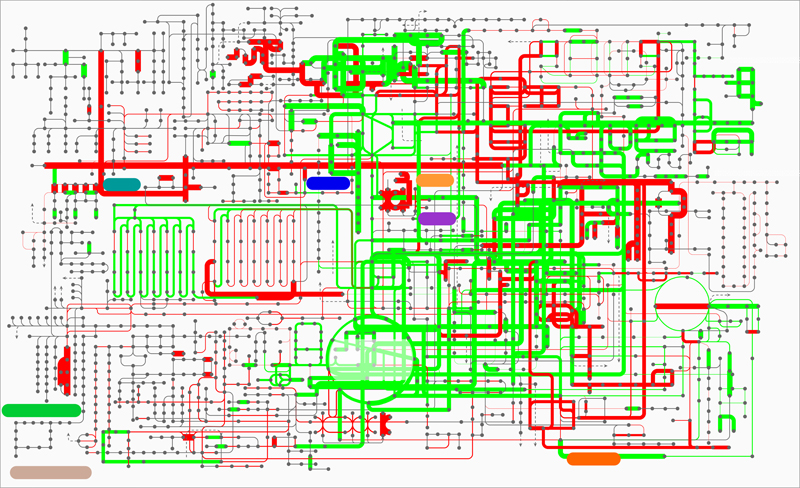


Figure S2. The significantly expressed genes mapped metabolic pathways in the D1 compared to DC group using iPATH2 online software. Red denotes up-regulation and green indicates down-regulation. Black indicates the control level. The significant level (*padj*-value) of genes is positively correlated with the edge width.


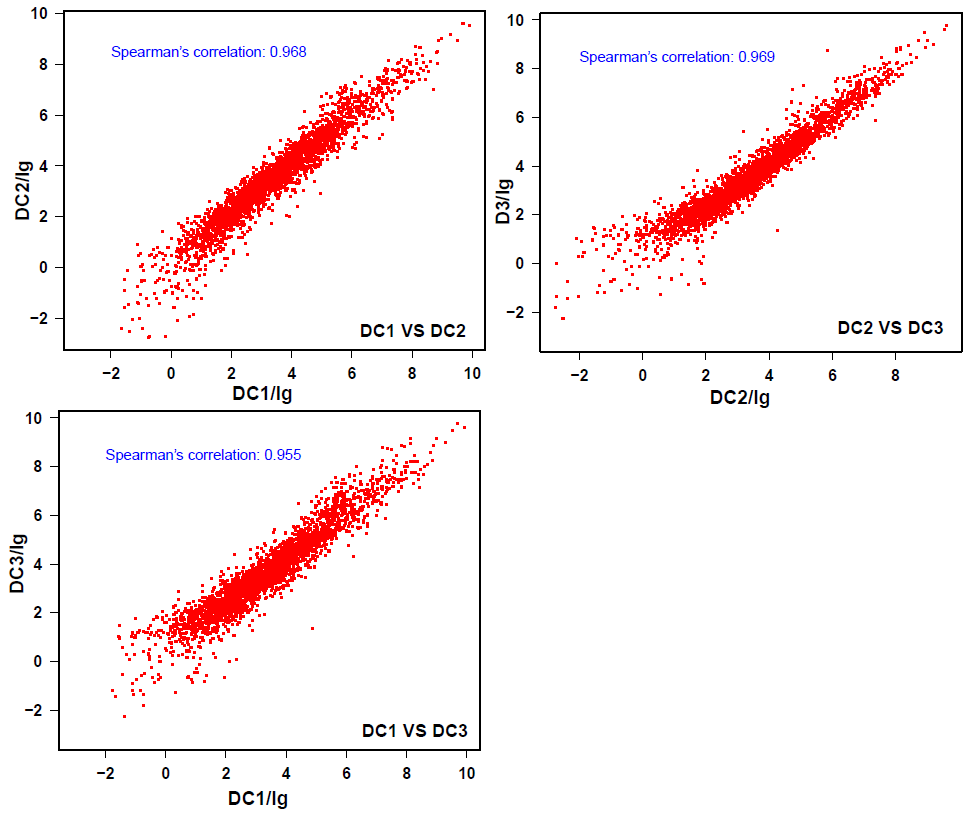


Figure S3. Representative inter-group correlation coefficients were calculated by the Spearman Rank-Order Correlation.

Figure S4. The representative total ion chromatogram of LC-MS based metabolomics for *D. radiodurans* R1 cell.


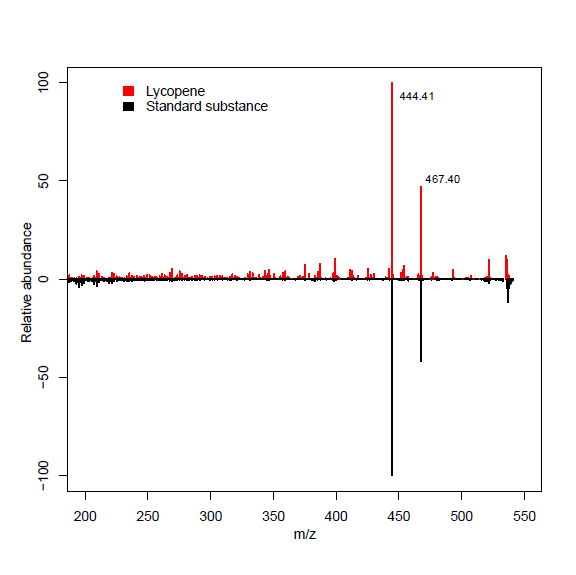


Figure S5. The positive ion MS/MS fragmentation of the lycopene from wild strains extracts (top panel) and the positive ion mode fragmentation spectrum for synthetic lycopene (bottom panel).


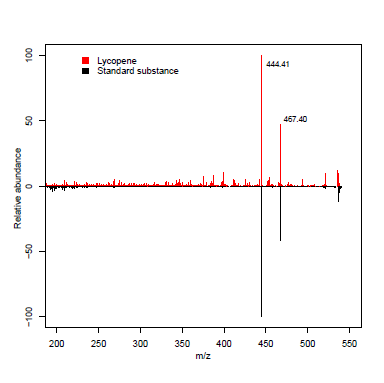


**References**

Li R, Li Y, Kristiansen K and Wang J (2008) SOAP: short oligonucleotide alignment program. Bioinformatics 24: 713-714.

Dillies MA, Rau A, Aubert J, Hennequet-Antier C, Jeanmougin M, et al. (2012) A comprehensive evaluation of normalization methods for Illumina high-throughput RNA sequencing data analysis. Brief Bioinform.

Anders S and Huber W (2010) Differential expression analysis for sequence count data. Genome Biol 11: R106.

Trapnell C, Williams BA, Pertea G, Mortazavi A, Kwan G, et al. (2010) Transcript assembly and quantification by RNA-Seq reveals unannotated transcripts and isoform switching during cell differentiation. Nat Biotechnol 28: 511-515.

Smith CA, Want EJ, O'Maille G, Abagyan R and Siuzdak G (2006) XCMS: processing mass spectrometry data for metabolite profiling using nonlinear peak alignment, matching, and identification. Anal Chem 78: 779-787.

Smith CA, O'Maille G, Want EJ, Qin C, Trauger SA, et al. (2005) METLIN: a metabolite mass spectral database. Ther Drug Monit 27: 747-751.

Wishart DS, Tzur D, Knox C, Eisner R, Guo AC, et al. (2007) HMDB: the Human Metabolome Database. Nucleic Acids Res 35: D521-526.

Su ZH, Zou GA, Preiss A, Zhang HW and Zou ZM (2010) Online identification of the antioxidant constituents of traditional Chinese medicine formula Chaihu-Shu-Gan-San by LC-LTQ-Orbitrap mass spectrometry and microplate spectrophotometer. J Pharm Biomed Anal 53: 454-461.

Rivera S, Vilaro F and Canela R (2011) Determination of carotenoids by liquid chromatography/mass spectrometry: effect of several dopants. Anal Bioanal Chem 400: 1339-1346.
